# Preparation and Characterization of Physicochemical Properties of Spruce Cone Biochars Activated by CO_2_

**DOI:** 10.3390/ma14143859

**Published:** 2021-07-10

**Authors:** Katarzyna Jedynak, Barbara Charmas

**Affiliations:** 1 Institute of Chemistry, Jan Kochanowski University, Uniwersytecka Str. 7, 25-406 Kielce, Poland; 2 Institute of Chemical Sciences, Faculty of Chemistry, Maria Curie-Sklodowska University, Maria Curie-Sklodowska Square 3, 20-031 Lublin, Poland; barbara.charmas@poczta.umcs.lublin.pl

**Keywords:** spruce cone, activated biochars, pyrolysis, physicochemical properties, ammonia adsorption

## Abstract

In this study the pyrolysis of Norway spruce cones, a lignocellulosic biomass was made. The biochar product was obtained by means of the physical activation method. CO_2_ was used as the activating gas. The surface properties of biochars were characterized by the nitrogen adsorption/desorption isotherms, scanning electron microscopy (SEM/EDS), X-ray fluorescence energy dispersion spectroscopy (ED-XRF), thermal analysis (TGA/DTA), infrared spectroscopy (ATR FT-IR), Raman spectroscopy and the Boehm’s titration method as well as the point of zero charge (pH_pzc_). The adsorption capacity and the possibility of ammonia desorption (TPD) were also examined. It has been shown that spruce cones can be successfully used as a cheap precursor of well-developed surface biochars, characterized by a large pore volume and good sorption properties. All obtained activated biochars exhibit a largely microporous structure and an acidic character surface. The investigated activated materials have the specific surface areas from 112 to 1181 m^2^ g^−1^. The maximum NH_3_ adsorption capacity of the activated biochar was determined to be 5.18 mg g^−1^ (88.22 mmol g^−1^) at 0 °C. These results indicate the applicability of spruce cones as a cheap precursor for the sustainable production of the cost-effective and environmentally friendly biochar adsorbent.

## 1. Introduction

The interest in using biochar in environmental protection has increased significantly due to its physical and chemical properties. Large surface area, low bulk density, great stability and strong adsorption capacity of biochar make it widely used in the sustainable environment and green technologies: monitoring of air pollution [1], wastewater treatment [2,3,4], biotechnology and renewable energy technologies as well as supercapacitors [5], catalysts [6,7] and green nanocomposites [8]. In addition, biochar plays an important role in improving soil fertility and increasing the carbon storage in the soil [9,10]. Preparation and application of biochar is one of the effective methods of waste recycling. It confines the unrenewable sources overexploitation and eliminates the disadvantage of high costs of such materials as conventional active carbon, graphene or carbon nanotubes [11].

Biochar is a fine-grained, porous, carbonaceous material produced in the process of biomass pyrolysis in an inert gas atmosphere [12,13,14]. Regardless of the solid fraction, carbonization generates also gas and liquid fractions which are potential energy sources [15]. It is characterized by a large number of surface groups and developed porosity due to the presence of micropores [16,17]. Moreover, biochars are highly carbonized materials and therefore they possess a great calorific value, comparable to that of high-rank coals [18].

The characteristics of biochar are largely dependent on the carbonization conditions [15]. High pyrolysis temperature often increases the surface area and the carbonized fraction which leads to a large adsorption capacity [19]. In addition, the properties of biochar are also influenced by the type of raw material from which it was obtained. Taking into account a wide range of available biomaterial wastes and different production techniques, a great variety of physicochemical properties of the obtained biochars should be expected [15].

For production of biochar there can be virtually used all types of biomass: sewage sludge [20], residues from the agricultural and food industries (e.g., rice husks, cotton stalks and nut shells [17,21,22], soybean husks [22], winter wheat at full maturity (grains and straw were pyrolyzed separately) and meadow grass [14], maple leaf [3], banana peels [23], cocoa tree (*Gliricidia sepium*) biomass [24], wastes of date palm [25] and oil palm shell [26], cork wastes [7], microalgae (*Spirulina* sp.) [2] and macroalgal (*Eucheuma spinosum*) biomass [4]), energy plants (e.g., corn cobs, poplar (*Populus*), willow (*Salix*) [14]) and raw materials of forest origin (e.g., tree bark [17,21,27,28,29]), eucalyptus residues [27], sphagnum moss [17,21,30,31], mature acorns (*Quercus pubescens*) and mature cypress cones (*Cupressus sempervirens pyramidals*) [32], pine cones [33,34], larch cones (*Larix decidua* Mill. Subsp. Decidua) and spruce (*Picea abies* LH Karst) [17], pine needles [9]) and many others.

Biochar obtained from the wastewater; the so-called sewage biochar is characterized by different properties from that derived from other raw, organic materials. Its carbon content is smaller which is not surprising given that the solids in the wastewater are composed of organic and inorganic substances while the biochar obtained from other biomass resources is composed mainly of organic matter and only a small amount of inorganic one. On the other hand, sewage biochar usually has a larger content of nitrogen, phosphorus and potassium, i.e., nutrients necessary for plant growth [15]. Unfortunately, they are also often characterized by a significant content of heavy metals, which is a potential source of water and soil contamination. However, in [35,36] it was shown that the risk of environmental contamination is smaller when using biochar than the starting sewage sludge.

Many scientists are increasingly interested in adsorbents with developed porosity. In order to develop the porous structure of the obtained biochars, the process of physical or chemical activation is applied. During the physical activation, the charred raw material is subjected to heat treatment at high temperatures in the presence of oxidizing agents such as carbon dioxide, water vapor or their mixture [37]. This type of activation was used in [20]. In the cited studies, the sewage sludge was activated with CO_2_ or air and the influence of different annealing times and temperatures was examined.

In turn, the chemical activation involves the carbonization of the precursor in the presence of chemical agents [38]. In this process, the raw material and the inorganic activating agents such as: KOH, NaOH, HNO_3_, ZnCl_2_ and H_3_PO_4_ are thoroughly mixed and then thermally activated in order to develop a porous structure [37,39]. This activation method was used in [20], KOH was mixed with the dried sewage sludge. The authors of the paper [26] used the chemical activation supported by the microwave energy. The obtained biochar was mixed with a potassium hydroxide solution at the biochar/KOH ratio of 1:1.75 (wt%). The other method of chemical activation is impregnation of the carbon precursors or the obtained carbon adsorbents with the solutions of the above-mentioned chemical compounds. The process of biomass impregnation was conducted with phosphoric acid (V) using various precursors, e.g., bark [40], pine cones [41], brown sphagnum [31]. On the other hand, treatment with the acids: HCl and HNO_3_ of mature acorns and cypress cones was applied in [32], while the activation of pine cones with NaOH was described in [33]. There were used two activation methods: physical mixing of the precursor with the activating agent or impregnation of the precursor with the NaOH solution, followed by different variants of thermal treatment at 600 °C. Özhan et al. [21] mixed the pine cones with the ZnCl_2_ solution using various proportions of impregnation to the weight at the concentrations: 10 to 100%.

Among the mentioned chemical compounds, alkali metal hydroxides are environmentally hazardous, expensive and corrosive, and ZnCl_2_ is unfriendly for the environment [42]. The increase in air pollution caused by such gases as ammonia (NH_3_) has proved to be a global problem [43]. Ammonia is a colorless, pungent and corrosive gas, which is also one of the most abundant nitrogen-containing compounds in the atmosphere, right after N_2_ and N_2_O [44]. NH_3_ is an important raw material for the production of nitric acid, ammonium sulphate, urea, plastics, rubbers, varnishes, bactericides and disinfectants. Ammonia is also used as a cooling gas in cooling installations. Actual ammonia emissions are mainly generated from industry (fertilizer and coke oven), fossil fuel combustion, cattle and poultry farming as well as cooling methods. Of all these sources the livestock waste management and production of fertilizers account for about 90% of the total ammonia emissions [45]. NH_3_ is a poisonous gas that affects not only the environment but also human health [44]. When the NH_3_ content in the atmosphere exceeds the range of 50–100 ppm, irritation of the eyes, throat and nose may occur [44,45]. The US Agency for Safety and Health at Work has set a limit of 50 ppm for 8 h of work. A long-term contact at the concentrations greater than 300 ppm may cause permanent injury or even death [44]. Given this, it is important to find an effective way to control ammonia emissions. Adsorption turned out to be the perfect solution to this problem. This process is effective even at low concentrations of pollutants, moreover, it enables any recovery of pollutants by desorption [43]. The adsorbents used for this process should be characterized by large surfaces areas and narrow pores [46].

The main aim of this study was to obtain cheap biochars from spruce cones as a precursor by activation with CO_2_ and characterize their physicochemical and adsorption properties (ammonia adsorption). The idea behind our research was to obtain cheap biochars with a developed microporous structure. Developing the microporosity of the obtained biochars was aimed at extending the possibilities of using this type of materials-first of all, concerning the adsorption capacity. The method of developing microporosity should significantly improve the parameters of the porous structure of the obtained biochars, i.e., increase their total specific surface area and total pore volume compared to the non-activated materials. Biochar obtained in this way can be used in the future, among others, for the removal of toxic gases (ammonia, carbon oxides (CO and CO_2_), sulfur oxides (SO_2_ and SO_3_) and nitrogen oxides (NO_x_)), as well as volatile organic compounds from the air. It is worth emphasizing that there are no data in the literature on obtaining biochar from spruce cones by physical activation with CO_2_, which is a novelty of this paper. Preliminary studies of the adsorption of gaseous ammonia on the obtained biocarbons were carried out in this paper.

## 2. Materials and Methods

### 2.1. Materials and Reagents

Norway spruce cones (*Picea abies* L. H. Karst) were used as a biochar precursor. The gases, ultra-high purity (UHP) nitrogen 5.0 (99.999%) and carbon dioxide 5.0 (99.998%) or 4.5 (99.995%) for preparation of biochars were purchased from Air Liquide (5.0) or Linde Gaz (4.5), Kielce, Poland. The gases, UHP helium 5.0 (99.999%) for cleaning (stabilization) of biochar and temperature-programmed desorption of ammonia (TPD), and UHP 10% ammonia in helium for pulse chemisorption were purchased from Air Liquide, Kielce, Poland.

### 2.2. Preparation of Biochars

Initially the samples of raw spruce cones were dried at 100 °C for 48 h. Then they were washed with the running and distilled water and next dried again for 24 h at a temperature of 100 °C. After this time the impurities were removed from the surface of the cones and the raw material was ground in a laboratory mill (MF 10, IKA, Staufen im Breisgau, Germany) to collect the grain size fraction of 0.5 mm.

The pyrolysis was made in a tube furnace (MRT-4, Czylok, Poland) in two ways:

- One-stage pyrolysis: raw material was heated from room temperature to 800 °C (heating rate: 5 °C min^−1^) in the nitrogen atmosphere (gas flow: 20 dm^3^ h^−1^). The material was kept at this temperature for 3 h. The obtained carbon was designated as BC-1.
- Three-step pyrolysis: the material was heated from room temperature to 180 °C (heating rate: 2 °C min^−1^). The precursor was kept at the temperature of 180 °C for 5 h. Then with the temperature rise rate of 5 °C min^−1^, the material was heated to 400 °C and kept for 1 h. The final temperature (obtained at the heating rate: 5 °C min^−1^), at which the material was soaked for 3 h, was 850 °C. The process proceeded in the nitrogen atmosphere at the rate of 20 dm^3^ h^−1^. The obtained carbon was designated as BC-2.

In order to develop the porous structure of the obtained biochars, the activation process was conducted. There were three variants used:

- The quartz boats with the sample BC-1 were placed in the furnace and heated from room temperature to 850 °C (heating rate: 10 °C min^−1^) in the nitrogen atmosphere (20 dm^3^ h^−1^). Then the activation process was conducted at this temperature by CO_2_ (99.998%) for 6 h (CO_2_ flow rate: 6 dm^3^ h^−1^). The cooling process was conducted in the nitrogen atmosphere (20 dm^3^ h^−1^). The obtained carbon was designated as BC-1-CO_2_-6h.
- The quartz boats with the sample BC-1 were placed in the furnace and heated from room temperature to 850 °C (heating rate: 10 °C min^−1^) in the nitrogen atmosphere (20 dm^3^ h^−1^). Then the activation process was carried out at this temperature by CO_2_ (99.995%) for 6 h or 12 h (CO_2_ flow rate: 6 dm^3^ h^−1^). The cooling process took place in the nitrogen atmosphere (20 dm^3^ h^−1^). The obtained carbons were designated as BC-1-CO_2_-6h* and BC-1-CO_2_-12h*.
- The quartz boats with the sample BC-2 were placed in the furnace and heated from room temperature to 850 °C (heating rate: 10 °C min^−1^) in the nitrogen atmosphere (20 dm^3^ h^−1^). Then the activation process was conducted at this temperature by CO_2_ (99.998%) for 6 h (CO_2_ flow rate: 6 dm^3^ h^−1^). The cooling process was conducted in the nitrogen atmosphere (20 dm^3^ h^−1^). The obtained carbon was designated as BC-2-CO_2_-6h.

**Figure 1:**
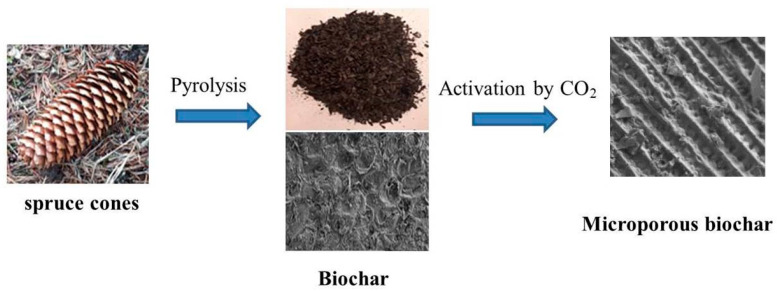
Preparation of microporous biochars.

Figure 1 shows the stages of microporous biochars preparation.

### 2.3. Characterization of Biochars

The biochars porous structure was determined from the low-temperature nitrogen adsorption/desorption isotherms (77 K) by means of the volumetric adsorption analyzer ASAP 2020 (Micromeritics, Norcross, GA, USA) (Structural Research Laboratory of Jan Kochanowski University in Kielce). The carbon samples were degassed at 473 K for 2 h before making the adsorptive measurements. The porous structure standard parameters: specific surface area, pore volume as well as pore size distribution were determined on the experimental nitrogen adsorption isotherms. Determination of the specific surface area (S_BET_) was made in the range of relative pressure from 0.05 to 0.20 taking into account the surface area (0.162 nm^2^) which was occupied by a single nitrogen molecule placed in the monolayer [47]. Determination of the total pore volume (V_t_) took place from one point of the adsorption isotherm which correspond to the relative pressure p/p_0_ = 0.99 [48]. To calculate the pore size distribution functions (PSDs), the non-local density functional (NLDFT) method was applied for the carbon slit-shaped pores characterized by the surface energetical heterogeneity and geometrical corrugation [49,50]. The calculations were made by applying the numerical program SAIEUS (Micromeritics).

The biochars morphology was studied by means of SEM Zeiss mod. Ultra Plus, EDS Bruker Quantax 400. A voltage of 2 kV was applied in the measurements. The energy-dispersive X-ray spectroscopy (SEM/EDX, acceleration: 15 kV) was used for the quantitative analyses.

For determination of the quantitative elemental composition of the biochars, there was applied the ED-XRF analysis (X-ray fluorescence energy dispersion spectroscopy). The measurements were performed in the Laboratory of Environmental Analytics (UJK) based on the Thermo Scientific’s Niton XL3t GOLDD +. The measurements accuracy was evaluated based on the POLISH VIRGINIA TOBACCO LEAVES (INCT-PVTL-6) as the reference material. The results from three independent measurements are expressed as the concentration in mg kg^−1^ or a percentage ± 2 SD.

The thermal stability of the activated carbons was assessed from the thermal analysis (Derivatograph-C, F. Paulik, L. Erdey, MOM, Budapest, Hungary). The measurements were performed in the air atmosphere and in the temperature range 20–800 °C whereby the temperature rise rate was 10 °C min^−1^. The reference material was Al_2_O_3_. The samples weight was about 30 mg. During the analysis there were recorded the TG, DTG and DTA curves.

A Perkin-Elmer Spectrum 400 FT-IR/FT-NIR spectrometer (Perkin-Elmer, Waltham, MA, USA) with a smart endurance single bounce diamond, attenuated total reflection (ATR) cell was used for infrared spectra recording. The 4000–650 cm^−1^ range spectra were obtained from the coaddition of 500 scans with a 4 cm^−1^ resolution. All samples were dried and powdered in an agate mortar before being measured.

The carbon matter structure in the biochar was studied making the Raman spectra by means of InVia Renishaw Raman spectrometer (Raman Station 400 F, Perkin Elmer, UK) applied with the microscope Leica, the thermoelectrically cooled CCD detector and argon laser (514.5 nm). There were the resolution of 1 cm^−1^ and accumulation of four spectra.

The Boehm’s titration method [51,52] was used for determination of the functional acidic and basic groups with oxygen on the carbon materials surface. The 0.2 g mass weights of the carbons were dispersed in the sodium bicarbonate, sodium carbonate, sodium hydroxide and sodium ethoxide solutions for determination of functional acidic groups. However, hydrochloric acid was used for determination of the total basic groups. Next the suspensions were shaken for 48 h at room temperature (Table 1). After the filtration of the samples, titration of 10 cm^3^ of the filtrate was made with 0.1 mol dm^−3^ HCl in order to determine acidic groups and with 0.05 mol dm^−3^ NaOH for the total basic group’s determination.

The carbon pH_pzc_ (point of zero charge) was analyzed by means of the method used in [53,54]. To begin with the 0.01 mol dm^−3^ NaCl solution was prepared. Then the pH was brought to that between 3 and 12 adding 0.1 or 1 mol dm^−3^ HCl and 0.1 or 1 mol dm^−3^ NaOH. The carbon materials samples were added to the solutions with a suitable pH value. Next, they were shaken in the incubator (Orbital Shaker—Incubator ES-20, Grant-bio) for 180 min at 298 K. Then there was measured the final pH. There were determined the relationships between the final and the initial values of pH. The pH_pzc_ indicates the intersection point of the experimental curves and pH_initial_ = pH_final_ line [53,54]. A pH-meter (inoLab pH 730, WTW) was used for measuring the pH value.

### 2.4. Pulse Chemisorption and Temperature-Programmed Desorption (TPD) of Ammonia

The measurements were made using the automatic AutoChem II 2920 analyzer (Micromeritics, Norcross, GA, USA). Before testing, all biochar samples were degassed at 200 °C for 2 h (ASAP 2020, Micromeritics, Norcross, GA, USA). Then the measurements were made using the Auto Chem II apparatus. At the beginning, each analyzed biochar sample (50 mg of biocarbon in a quartz reactor) was cleaned (stabilized) in the helium atmosphere at a temperature of 250 °C (20 °C min^−1^ for 40 min).

Chemisorption studies were carried out at three temperatures: 0 °C, 10 °C and 20 °C. The standard gas (10% NH_3_ in helium) was dosed from a loop of known volume. The TCD detector registered the next doses. Dosing was performed until the surface of the analyzed material was saturated. The total amount of gaseous ammonia adsorbed on the biochar surface was calculated. In the next stage, the thermo programmed desorption experiment was performed. The temperature was increased from the measurement temperature at which the chemisorption process was initially carried out, to 250 °C (with a temperature increase rate 10 ° C min^−1^).

## 3. Results and Discussion

### 3.1. Characterization of Biochars

#### 3.1.1. Porous Structure of Biochars

**Figure 2:**
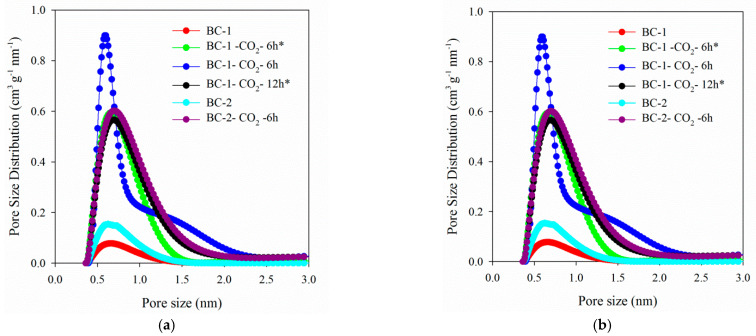
Nitrogen adsorption/desorption isotherms (**a**) and pore size distribution curves (**b**) for the studied biochar materials.

Figure 2a,b present the nitrogen adsorption/desorption isotherms and pore size distributions for the studied biochar materials. Based on the IUPAC classification [55], it can be concluded that all registered isotherms (Figure 2a) have the shape of the type I reversible isotherm. These isotherms show clear adsorption at low relative pressures which in turn, proves largely developed microporosity. The mesoporosity share is insignificant—the course of isotherms in the range of medium and high relative pressures is almost (or completely) parallel to the axis of relative pressures. All the isotherms have the H4 type hysteresis loops, the existence of which is mainly attributed to the materials with narrow pores.

When analyzing the pore volume distribution functions for the tested biochars, it is observed that each of the presented curves contains one maximum which corresponds to the micropores. The heights of the respective peaks and their dispersion are different for the tested bio-carbons—they indicate the micropores share in the total porosity.

When analyzing the distribution functions for the tested biochars, the largest proportion of micropores was found for the material BC-1-CO_2_-6h (highest peak), and the smallest for the unactivated biochar created as a result of one-stage BC-1 carbonization (lowest peak). Undoubtedly, the higher peaks indicate the development of microporosity as a result of the activation process with CO_2_. The exact values of the micropore dimensions w_mi_ determined from the maximum of the DFT distribution function are presented in Table 1.

Table 1 presents the standard parameters of the porous structure for the tested carbon materials determined on the basis of the experimental nitrogen adsorption isotherms. Among all the obtained carbon materials, the BC-1-CO_2_-6h (1181 m^2^ g^−1^) had the largest specific surface area (S_BET_). In order to determine the influence of the activation process on the porous structure of the biochars, the porous structure parameters were also determined for the biochar before the activation process. The non-activated biochars were characterized by significantly smaller S_BET_ values (BC-1: 112 m^2^ g^−1^ and BC-2: 206 m^2^ g^−1^). The biochars obtained as a result of the 12-h activation possess a smaller specific surface area in comparison to the materials activated for 6 h after the three-stage pyrolysis. In addition to the carbonization method, this result can be also influenced by the smaller purity of CO_2_ used to obtain the BC-1-CO_2_-12h*. A similar relationship can be observed for the BC-1-CO_2_-6h* and BC-1-CO_2_-6h carbon materials (S_BET_: 884 and 1181 m^2^ g^−1^, respectively). Comparing the obtained values of S_BET_ with those presented in [21,33] where activated biochars were obtained from pine cones, it can be noticed that in this study the S_BET_ values are much higher in some cases.

Analyzing the results presented in Table 1 one can observe that after activation all biochars have a developed porosity, and what is important, micropores dominate in their structure. After activation there was an increase in the total pore volume (V_t_) compared to that of the non-activated biochar. The BC-1-CO_2_-6h carbon (0.60 cm^3^ g^−1^) is characterized by the largest V_t_ value. Before activation the carbon materials had significantly smaller values (0.08–0.14 cm^3^ g^−1^). The BC-1-CO_2_-6h biochar is also characterized by the largest volume of micropores (0.43 cm^3^ g^−1^).

#### 3.1.2. SEM/EDS Analysis

**Figure 3:**
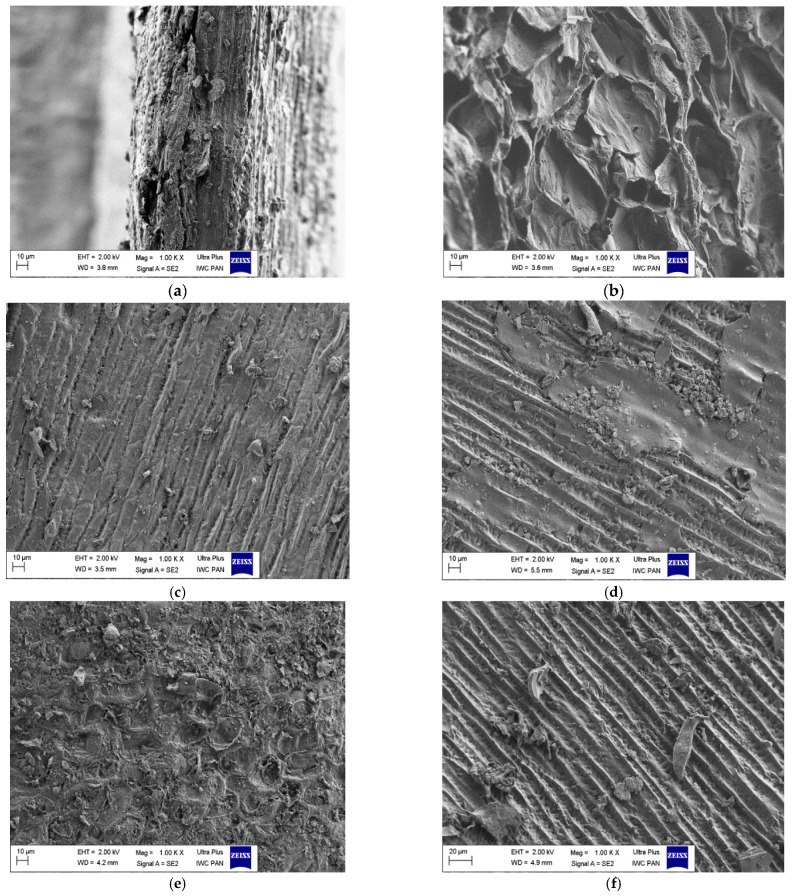
SEM photos of the obtained biochar: (**a**) BC-1; (**b**) BC-1-CO_2_-6h*; (**c**) BC-1-CO_2_-6h; (**d**) BC-1-CO_2_-12h*; (**e**) BC-2; (**f**) BC-2-CO_2_-6h.

Figure 3a–f shows the SEM pictures for the tested biochars. The SEM images present the differences in the morphology of the obtained biochars. After the activation process, almost all of the tested biochars are characterized by a layered structure, which proves their order. The layered structure is visible for the carbons: BC-1-CO_2_-6h, BC-1-CO_2_-12h* and BC-2-CO_2_-6h (Figure 3c,d,f). The SEM picture for the BC-1-CO_2_-6h* carbon shows a different arrangement from those of the other activated carbons (Figure 3b). After the activation process all tested carbons are characterized by the developed porosity. The unactivated biochars are observed to be much less porous (Figure 3a,e).

Simultaneously with the SEM analysis, the EDS (X-ray energy dispersion spectroscopy) studies were carried out. This technique allows for the qualitative and quantitative elemental analyses of the studied material [56]. The results of EDS chemical composition microanalysis for the obtained biochars are presented in Table 2. When analyzing the obtained results, it can be found that the basic chemical components of biochar are carbon and oxygen, and in most cases: calcium, magnesium and potassium. The presence of C and O results most likely from the presence of lignin, cellulose and hemicellulose in the cones [57]. The amount of carbon in the obtained biocarbons ranges from 80.09 to 89.05% *w/w*. The oxygen content is from 6.56 to 10.85% *w/w*. The presence of other elements depends largely on the chemical composition of the biomass (Table 3).

#### 3.1.3. ED-XRF Analysis

Table 3 shows the results of the ED-XRF analysis of a raw cone and for example, of the selected BC-2 material. The presence of most elements was confirmed in both methods (Table 2 and Table 3). The presence of such elements as Cl, Fe, Rb, Sr, Zn and Zr was confirmed by the ED-XRF method, which was not confirmed by the SEM-EDX method.

#### 3.1.4. Thermal Analysis

**Figure 4:**
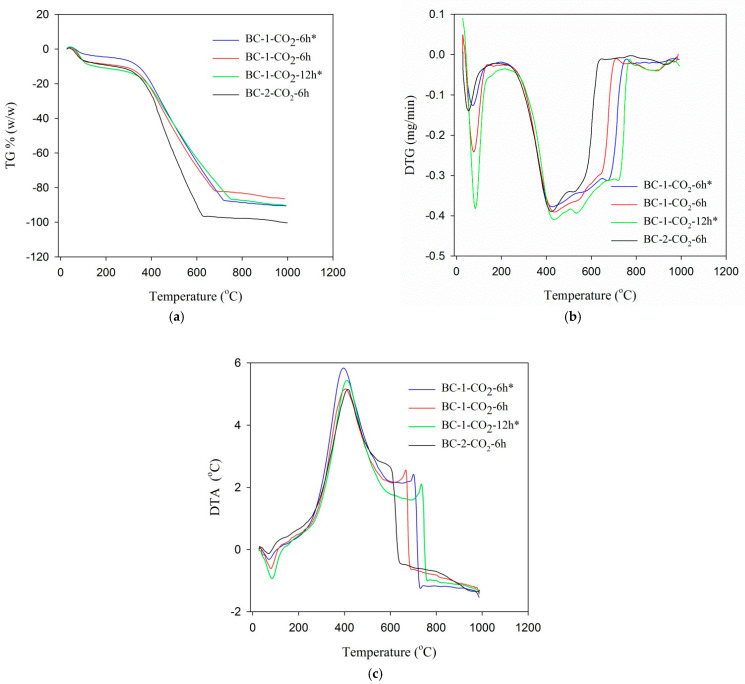
Course of the TG% (**a**), DTG (**b**) and DTA (**c**) curves for studied biochars.

Figure 4a–c shows the TG, DTG and DTA curves for the studied activated biochars. All tested activated biochars are characterized by a large thermal stability (Figure 4a). There are three main stages of weight loss. The first stage takes place at a temperature of approximately 150 °C (maximum weight loss is at approximately 105 °C). In the thermograms single peaks related to the desorption of physically bound water (moisture) can be observed. In the second stage, from 250 °C to 420 °C, the greatest weight loss occurs due to the pyrolysis of cellulose, hemicellulose and part of lignin [14,28]. In the third step from 430 °C to 850 °C, there is also a significant mass loss. In this temperature range, the biomass thermooxidative degradation processes are complex (Figure 4b) and include: the reaction of lignin decomposition at a higher temperature (which is the main reaction), the decomposition of inorganic materials present in the spruce cones and the slow carbonization of the created carbon [58]. Lignin is pyrolysed to tar and then at a higher temperature secondary cracking occurs and volatile gas is released [57]. The least ordered materials undergo thermal degradation the fastest. As can be seen from Figure 4a–c the most intense degradation occurs in the case of BC-2-CO_2_-6h. The complete carbonization process is over at 610 °C while the complete decomposition of more ordered materials (BC-1-CO_2_-6h, BC-1-CO_2_-6h* and BC-1-CO_2_-12h) occurs at higher temperatures (700–800 °C).

#### 3.1.5. ATR-FTIR Analysis

**Figure 5:**
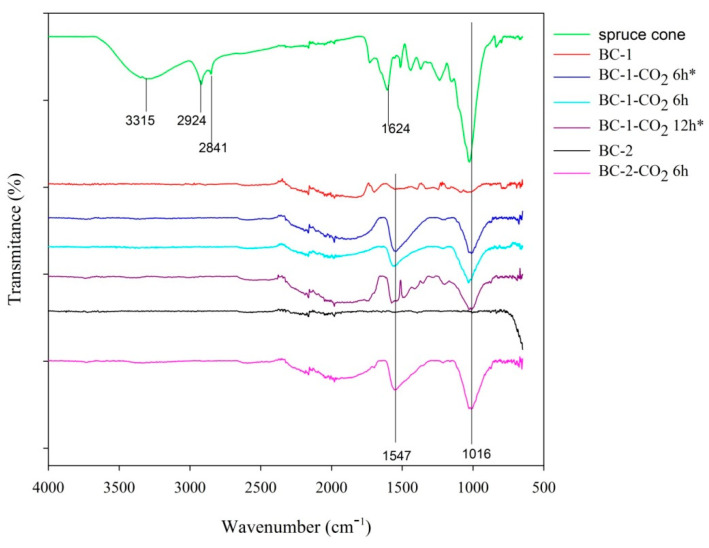
FTIR spectra of the studied spruce cone, unactivated and activated biochars.

The ATR-FTIR spectra are presented in Figure 5. The spectrum of spruce cones shows the strong absorption bands located at 3315 cm^−1^, 2924 cm^−1^, 2841 cm^−1^, 1624 cm^−1^ and 1016 cm^−1^. The first of these bands corresponds to the asymmetric and symmetric stretching vibrations of O-H of the water molecule [59,60]. The presence of water is also associated with the band at 1624 cm^−1^. The absorption in the range of 3000–2800 cm^−1^, exactly the bands at 2924 cm^−1^ and 2841 cm^−1^, is characterized by the asymmetric stretching vibrations of C-H bonds of alkyl groups (-CH_3_, -CH_2_) [61]. On the other hand, the band at 1016 cm^−1^ corresponds to the C-O stretching vibrations, which most often occur in carbohydrates [59].

In the case of the tested non-activated and activated biochars, the IR bands occur at 1547 cm^−1^ and 1016 cm^−1^. The band located at 1547 cm^−1^ is characterized by the C=O stretching vibrations of the carbonyl group [60]. The presence of this group on the surface of the tested biochars is consistent with the Boehm titration results presented below (Section 3.1.7). As in the case of the starting material-spruce cones, the band at 1016 cm^−1^ is related to the C-O stretching vibrations [59].

#### 3.1.6. Raman Analysis

**Figure 6:**
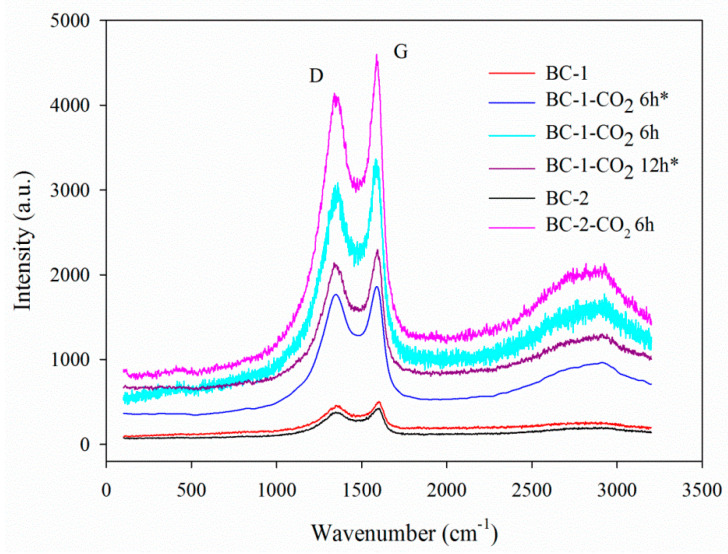
Raman spectra of the unactivated and activated biochars.

The Raman spectra of the studied biochars are presented in Figure 6. The curves exhibit two broad overlapped bands with the maxima at 1360 cm^−1^ and 1590 cm^−1^, which correspond to the D and G bands, respectively, indicating the extent of graphite disorder. The G band at 1590 cm^−1^ indicates the stretching vibrations of sp^2^ bonds in the aromatic, hexagonal graphene planes [62]. When biochars are disordered (Figure 6), two broad peaks appear, because of sp^3^ (at 1360 cm^−1^, D band) and sp^2^ (1590 cm^−1^, G band) structures vibration. The bands in Figure 6 are relatively broad, indicating the presence of small graphite crystallite sizes and the turbostratic structures of the biochars.

#### 3.1.7. Boehm’s Titration Method

The Boehm’s titration method [51,52] was used to determine the chemical nature of biochars surface. This method involves carrying out the reaction of the exchange of H^+^ ions into Na^+^ ions from the bases of different power, i.e., different pK. The individual acid groups succumb neutralization in the following order:

(1) Carboxylic-affected by NaHCO_3_.
(2) Carboxyl + lactone-affected by Na_2_CO_3_.
(3) Carboxylic + lactone + phenolic-affected by NaOH.
(4) Carboxylic + lactone + phenolic + carbonyl-affected by C_2_H_5_ONa.

Summing up, the content of basic functionalities was determined by titration of unreacted HCl that was previously used to neutralize the basic groups, with NaOH solution.

Table 4 shows the functional groups determined on the surface of the studied biochars. All materials tested in this study are characterized by both acidic and basic properties, however, basic properties prevail. In the acid groups determined by the Boehm’s method, the presence of only carbonyl groups was confirmed. In these groups the carbon BC-2-CO_2_-6h is characterized by the largest concentration. The FTIR method confirmed the presence of carbonyl groups for all tested carbons, however, for the BC-2-CO_2_-6h carbon, the largest intensity of the band corresponding to the C=O group is apparent.

#### 3.1.8. The Point of Zero Charge (pH_pzc_)

**Figure 7:**
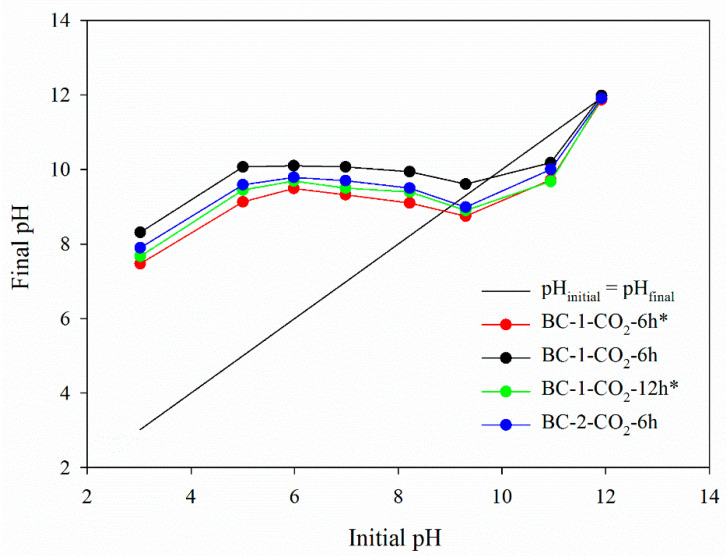
pH_pzc_ for studied biochars (pH drift method).

The pH_pzc_ determines the pH value at which the net surface charge on the adsorbent equals zero [53,63]. The pH drift method results for the studied biochars are presented in Figure 7. The values of pH_pzc_ determined from the graph are as follows: for BC-1-CO_2_-6h*: 8.88; for BC-1-CO_2_-6h: 9.75; for BC-1-CO_2_-12h* 9.01; for BC-2-CO_2_-6h: 9.07. This indicates that in the solution of pH> pH_pzc_ the biochar surface has a negative charge while for pH <pH_pzc_ the surface has a positive charge [53,63].

### 3.2. Pulse Chemisorption and Temperature-Programmed Desorption (TPD) of Ammonia

Table 5 presents the results of the study of pulse chemisorption and temperature-programmed desorption (TPD) of ammonia for the tested biochars. The results show that the ammonia adsorption process is significantly influenced by the measurement temperature (Table 5). The dependence resulting from different variants of activation with CO_2_ was observed. Probably the adsorption properties of the biochars are significantly affected not only by the temperature of the process but also the purity of the gas (CO_2_) used during the activation process (see Section 2.2 Preparation of Biochars). In the case of two biochars obtained according to Variant II of the activation, the adsorption capacity increases with the temperature increase. Such a relationship was found for the biochars: BC-1-CO_2_-6h* and BC-1-CO_2_-12h* where the maximum adsorption capacity at 20 °C was: 2.69 mmol g^−1^ and 3.59 mmol g^−1^, respectively. A different relationship was observed in the case of the biochars obtained according to activation variants I and III. This applies to the two biochars, i.e., BC-1-CO_2_-6h and BC-2-CO_2_-6h, the maximum values of the adsorption capacity at 0 °C were: 5.18 mmol g^−1^ and 3.95 mmol g^−1^, respectively. The porous structure has also a significant impact on the adsorption properties of the obtained biochars. The dependence of the influence of the porous structure parameters on the adsorption properties of the tested biochars was observed (Table 1), the larger the specific surface area, and thus the more developed the porous structure of the tested adsorbents, the better the ammonia adsorption properties. For example, the BC-1-CO_2_-6h biochar (5.18 mmol g^−1^; 0 °C) has the largest ammonia adsorption capacity. This material has the largest specific surface of the analyzed biochars (1181 m^2^ g^−1^).

Analyzing the ammonia TPD data for the tested biochars (Table 5), it was observed that the tested carbon materials adsorbed a relatively large amount of ammonia while its small part was desorbed. Taking this into account, it can be concluded that the analyzed biochars are promising as storage materials for the environmentally harmful gas, which is ammonia.

Table 6 compares the maximum adsorption capacity (biochars and activated carbons) used for the adsorption of ammonia from the gas phase. It was observed that the adsorbents tested in this paper are characterized by good properties in comparison to those of other carbon materials.

## 4. Conclusions

The physicochemical properties of bio-carbons obtained in the activation process were investigated. It was shown that spruce cones can be successfully used as an economical precursor for the production of effective carbon adsorbents with very good parameters of the porous structure: large specific surface area and pore volume resulting in large adsorption capacity. The thermal treatment variant and the activation method have a significant influence on the physicochemical and adsorptive properties. The research proved that the biocarbons obtained as a result of activation have a very well-developed microporosity and are characterized by the partially crystalline structure. Unactivated biochars are characterized by less developed specific surface areas and much smaller pore volumes. It was shown that activation had also a positive effect on the ordering of the porous structure and as a result, the materials with an ordered porous structure were obtained. All tested activated biochars are characterized by significant thermal stability. During the determination of surface oxygen functional groups for all biochars there was observed the presence of both acidic and basic groups with a remarkable predominance of basic ones. The present study proved that the biochar based on spruce cones was an effective adsorbent for removing ammonia from the vapor phase.

## Figures and Tables

**Table 1:** Structural parameters of the studied biochars.

Biochar Materials	S_BET_ (m^2^ g^−1^)	V_t_ (cm^3^ g^−1^)	V_ultra_^DFT^ (cm^3^ g^−1^)	V_micro_^DFT^ (cm^3^ g^−1^)	V_meso_ (cm^3^ g^−1^)	w_mi_^DFT^ (nm)
BC-1	112	0.05	0.02	0.04	0.01	0.65
BC-1-CO_2_-6h*	884	0.38	0.13	0.33	0.05	0.71
BC-1-CO_2_-6h	1181	0.60	0.17	0.43	0.17	0.59
BC-1-CO_2_-12h*	1049	0.50	0.12	0.38	0.12	0.68
BC-2	206	0.08	0.03	0.07	0.01	0.63
BC-2-CO_2_-6h	1167	0.56	0.13	0.42	0.14	0.70

S_BET_—the specific surface area, V_t_—the total (single-point) pore volume obtained from the amount adsorbed at p/p_0_ ≈ 0.99, V_ultra_^DFT^—the ultramicropores volume (pores < 0.7 nm) obtained on the basis of DFT PSD, V_micro_^DFT^—the micropores volume (pores < 2 nm) obtained on the basis of DFT PSD, V_me_—the mesopores volume (pores 2–50 nm) obtained from the difference of V_t_ and V_mi_.

**Table 2:** Results of the EDS chemical composition microanalysis.

Elements	BC-1 (% *w/w*)	BC-1-CO_2_-6h* (% *w/w*)	BC-1-CO_2_-6h (% *w/w*)	BC-1-CO_2_-12h* (% *w/w*)	BC-2 (% *w/w*)	BC-2-CO_2_-6h (% *w/w*)
C	85.26	89.05	84.22	80.09	82.88	86.82
O	10.44	9.67	10.85	6.56	10.78	7.91
Ca	1.37	0.26	0.97	-	1.08	0.69
Mg	0.18	0.11	0.19	-	0.18	0.15
K	2.53	0.16	2.87	12.61	0.11	3.86
Al	-	-	-	-	0.17	0.06
Si	0.22	-	-	-	3.92	0.12
S	-	-	0.33	-	-	0.11
P	-	0.07	0.57	0.74	0.11	0.28
F	-	0.68	-	-	0.77	-

**Table 3:** Results of the ED-XRF analysis.

Elements	Spruce Cone	BC-2
Ca (%)	0.432 ± 0.02	1.51 ± 0.03
K (%)	1.41 ± 0.02	5.57 ± 0.04
P (%)	0.132 ± 0.077	0.50 ± 0.02
S (%)	0.20 ± 0.01	0.20 ± 0.01
Si (%)	0.27 ± 0.02	0.51 ± 0.03
Cl (mg/kg)	492 ± 20	312 ± 27
Fe (mg/kg)	71 ± 15	985 ± 35
Rb (mg/kg)	3 ± 1	22 ± 1
Sr (mg/kg)	6 ± 1	25 ± 1
Zn (mg/kg)	79 ± 5	<15
Zr (mg/kg)	4 ± 1	9 ± 1

**Table 4:** Oxygen functional groups on the surface of the studied biochars.

Adsorbents	Total Basic Groups (mmol g^−1^)	Total Acidic Groups (mmol g^−1^)	Phenolic Groups (mmol g^−1^)	Lactone Groups (mmol g^−1^)	Carboxylic Groups (mmol g^−1^)	Carbonyl Groups (mmol g^−1^)
**BC-1**	0.44	0.22	not determined ^1^	not determined ^1^	not determined ^1^	0.22
BC-1-CO_2_-6h*	1.04	0.67	not determined ^1^	not determined ^1^	not determined ^1^	0.67
**BC-1-CO_2_-6h**	1.33	0.78	not determined ^1^	not determined ^1^	not determined ^1^	0.78
BC-1-CO_2_-12h*	1.07	0.70	not determined ^1^	not determined ^1^	not determined ^1^	0.70
**BC-2**	0.51	0.26	not determined ^1^	not determined ^1^	not determined ^1^	0.26
**BC-2-CO_2_-6h**	1.12	0.75	not determined ^1^	not determined ^1^	not determined ^1^	0.75

Not determined ^1^–means that the groups marked in the table were not detected by the analytical method. *: the biochars activation process was carried out by CO_2_ (99.995%).

**Table 5:** Ammonia capacity and TPD for the studied biochars.

Biochars	NH_3_ Adsorption Capacity (mmol g^−1^) Pressure ~750 mmHg			TPD NH_3_ (mmol g^−1^) Pressure ~750 mmHg		
**T (°C)**	**0**	**10**	**20**	**0–250**	**10–250**	**20–250**
BC-1-CO_2_-6h*	1.06	2.33	2.69	0.36	0.32	0.23
**BC-1-CO_2_-6h**	5.18	3.39	1.13	0.20	0.24	0.30
BC-1-CO_2_-12h*	0.82	1.01	3.59	0.37	0.20	0.23
**BC-2-CO_2_-6h**	3.95	2.99	0.95	0.46	0.32	0.14

*: the biochars activation process was carried out by CO_2_ (99.995%).

**Table 6:** Comparison of the maximum adsorption capacity of the prepared biochars with that of other adsorbents.

Carbon Materials	S_BET_ m^2^ g^−1^	Adsorption Capacity (mg g^−1^)	Adsorption Capacity (mmol g^−1^)	Ref.
BC-1-CO_2_-6h*	884	18.05–45.81	1.06–2.69	This study
**BC-1-CO_2_-6h**	1181	19.24–88.22	1.13–5.18	This study
BC-1-CO_2_-12h*	1049	13.97–61.14	0.82–3.59	This study
**BC-2-CO_2_-6h**	1167	16.18–67.27	0.95–3.95	This study
**AC ^1^**	1161	20.27	1.19	[64]
**Na-OH-AC ^2^**	1125	28.78	1.69	[64]
**HNO_3_-AC ^3^**	1010	52.28	3.07	[64]
**H_2_SO_4_-AC ^4^**	1016	47.34	2.78	[64]
**AA-WS250-AR ^5^**	851	53.09	3.11	[65]
**OAK-250-KOH ^6^**	-	25	1.47	[66]
**OAK-250-H_2_O_2_^7^**	-	25	1.47	[66]
**OAK-450-KOH ^8^**	-	6	0.35	[66]
**OAK-450-H_2_O_2_^9^**	-	10	0.59	[66]
**AC ^10^**	430	13.28–71.36	0.78–4.19	[67]
**AC ^11^**	450	13.11–86.51	0.77–5.08	[67]

^1^ Active carbon pellets (Kanto Kagaku); ^2^ Active carbon was impregnated with NaNO_3_ in water, calcined in the He flow at 773 K for 3 h; ^3,4^ Active carbons were activated by the aqueous HNO_3_ or H_2_SO_4_ solution and finally calcined in the He flow at 773 K for 3 h.; ^5^ biochars obtained from the wood shaving waste, pirolysis at 250 °C, activation with 30% H_3_PO_4_ (450 °C/60 min); ^6^ hydrochar obtained from the oak wood, pirolysis at 250 °C, activation with KOH; ^7^ hydrochar obtained from the oak wood, pirolysis at 250 °C, activation with H_2_O_2;_^8^ biochar obtained from the oak wood, pirolysis at 450 °C, activation with KOH; ^9^ biochar obtained from the oak wood, pirolysis at 450 °C, activation with H_2_O_2_; ^10^ activated carbon (Aldrich Darco), purchased from Sigma-Aldrich Co.; ^11^ activated carbon (Merk), purchased from Merck KGaA. *: the biochars activation process was carried out by CO_2_ (99.995%).

## Data Availability

Not applicable.
